# Transcriptional enhancement of Smn levels in motoneurons is crucial for proper axon morphology in zebrafish

**DOI:** 10.1038/srep27470

**Published:** 2016-06-07

**Authors:** Zoltán Spiró, Angela Koh, Shermaine Tay, Kelvin See, Christoph Winkler

**Affiliations:** 1 Department of Biological Sciences, National University of Singapore, Singapore; 2 NUS Centre for BioImaging Sciences (CBIS), Singapore

## Abstract

An unresolved mystery in the field of spinal muscular atrophy (SMA) is why a reduction of the ubiquitously expressed Smn protein causes defects mostly in motoneurons. We addressed the possibility that this restricted vulnerability stems from elevated Smn expression in motoneurons. To explore this, we established an *ex vivo* zebrafish culture system of GFP-marked motoneurons to quantitatively measure Smn protein and *smn* mRNA levels as well as promoter activity in motoneurons versus other cell types. Importantly, we uncovered that Smn levels are elevated in motoneurons by means of transcriptional activation. In addition, we identified the ETS family transcription factor Etv5b to be responsible for increased *smn* transcription in motoneurons. Moreover, we established that the additional supply of Smn protein in motoneurons is necessary for proper axonogenesis in a cell-autonomous manner. These findings demonstrate the reliance of motoneurons on more Smn, thereby adding a novel piece of evidence for their increased vulnerability under SMA conditions.

SMA is the most common genetic cause of infant death and is characterized by the degeneration of lower α-motoneurons and subsequent muscle atrophy^1^. Reduced levels of Survival of Motor Neuron (Smn), a protein controlling snRNP assembly, are responsible for SMA^2^,^3^,^4^. Such reduction appears to be tolerable for most cells, while motoneurons are impaired. In addition, Schwann cells and neurons of the enteric nervous system are also affected in the SMA mouse model^5^,^6^, explaining why a motoneuron specific replenishment of Smn is only sufficient to restore functionality of the motor unit, but does not improve survival of SMA zebrafish and mice^7^,^8^,^9^,^10^,^11^,^12^. These findings suggest that cell-autonomous and non-cell-autonomous mechanisms together contribute to SMA. By complementing studies in mice, zebrafish offers a powerful system to understand the etiology of SMA. Accordingly, knockout or knockdown of *smn* in zebrafish embryos using genetic mutations, morpholino oligonucleotides (MO) or synthetic micro-RNAs results in developmental defects including morphological abnormalities of motoneuron axons^13^,^14^,^15^,^16^.

To explain the increased dependence of motoneurons on Smn, current hypotheses propose that motoneurons are more vulnerable to reduced splicing efficiency^17^,^18^,^19^, and/or that Smn is necessary for axonal localization of various mRNAs^20^,^21^,^22^,^23^. These models raise the possibility that motoneurons might express more Smn protein to satisfy an extra demand for this protein. Based on this, we thought to address the increased sensitivity of motoneurons in SMA by a novel hypothesis: are Smn levels indeed elevated in motoneurons compared with other cells, and if so, is such additional supply of Smn essential for their axonal development? Although previous studies had suggested that Smn levels are elevated in the motoneuron-rich ventral horn of rat and human spinal cords^24^,^25^, this question has never been addressed quantitatively at the single-cell level.

## Results and Discussion

**Figure 1:**
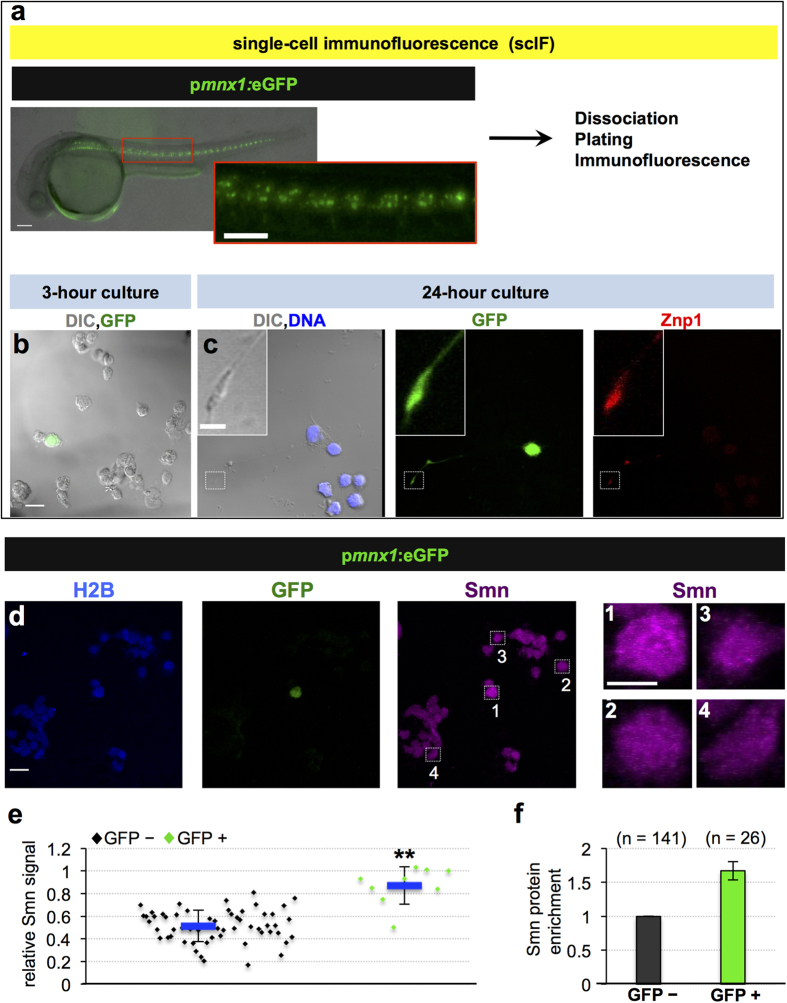
Motoneurons exhibit elevated Smn levels. (**a–c**) Single-cell immunofluorescent (scIF) experiments. 24 hpf p*mnx1*:eGFP embryos were dissociated and immunostained for Smn ((**a**) see also Methods). DIC image of cells following 3-hour (**b**) and 24-hour (**c**) incubation are shown with GFP in green. Note that the GFP signal in the cell body is oversaturated so that the weaker signal in the axon becomes visible. In (**c**) DNA is in blue and the motoneuron marker Znp1 (synaptotagmin) is in red; the axon growth cone is magnified in the corner of the images. Scale bars are 100 μm (**a**), 10 μm (low magnification in (**b**,**c**)) and 2 μm (high magnification in (**c**)). (**d**) scIF on p*mnx1*:eGFP embryos. Histone 2B (H2B), GFP and Smn signals are shown in Z-projected confocal sections. Cells marked by white rectangles are magnified on the right. Scale bars: 10 μm for low and 5 μm for high magnification. (**e**) To account for potential variability in the immunostaining, Smn levels were quantified relative to H2B. Diamonds denote GFP negative (GFP−) and GFP positive (GFP+) cells from one representative experiment. Blue bars indicate mean ± SD with significance values of *p < 0.05 and **p < 0.01. Exact values are (mean ± SD) 0.51 ± 0.14 (GFP−) and 0.87 ± 0.17 (GFP+), p = 0.001 with Wilcoxon Sum Rank Test. For more details, see Materials and Methods and Supplementary Table S1. (**f**) Average increase of Smn levels in motoneurons versus control cells. The exact value of enrichment is (mean ± SD): 1.67 ± 0.14. N = 3 experiments, n = number of analyzed cells.

To assess the levels of Smn protein in motoneurons, we established an *ex vivo* culture system of dissociated zebrafish embryos coupled with Smn immunofluorescence (single-cell immunofluorescence, scIF; Fig. 1a–c). Motoneurons were identified by eGFP expression under control of the motoneuron-specific *mnx1/hb9* promoter, allowing for direct comparison of Smn levels in motoneurons versus other cells. GFP positive cells developed Znp-1 positive axons after overnight incubation confirming their neuronal identity (Fig. 1c). The quantification of Smn levels was performed on cells fixed shortly after dissociation (see Methods for details). The specificity of the used Smn antibody was confirmed by scIF of *smn* MO injected embryos (Fig. S1a–e) and by Western blot analysis (Fig. S1f). Moreover, pre-adsorbing the antibody with the N-terminal part of human SMN resulted in almost no detectable signal in the scIF assay (Fig. S1g–k). Intriguingly, comparing Smn levels in GFP-positive and GFP-negative cells revealed that motoneurons contain on average 70% more Smn than other cells (Fig. 1d–f). This increase was not due to a difference in cell sizes, as motoneurons and surrounding cells have comparable sizes (Fig. S2a,b). We similarly observed slightly elevated levels of Smn in motoneuron cell bodies in cryo-sectioned intact spinal cords (Fig. S1l). To test whether such elevation is specific to motoneurons, we performed scIF in *nkx2.2a*:GFP embryos marking perineurial cells that originate from the ventral spinal cord and ensheath the motor nerve^26^. We found no difference in Smn levels of those cells compared to control cells (Fig. S2c–e), suggesting that the elevation of Smn levels is a hallmark of motoneurons.

**Figure 2:**
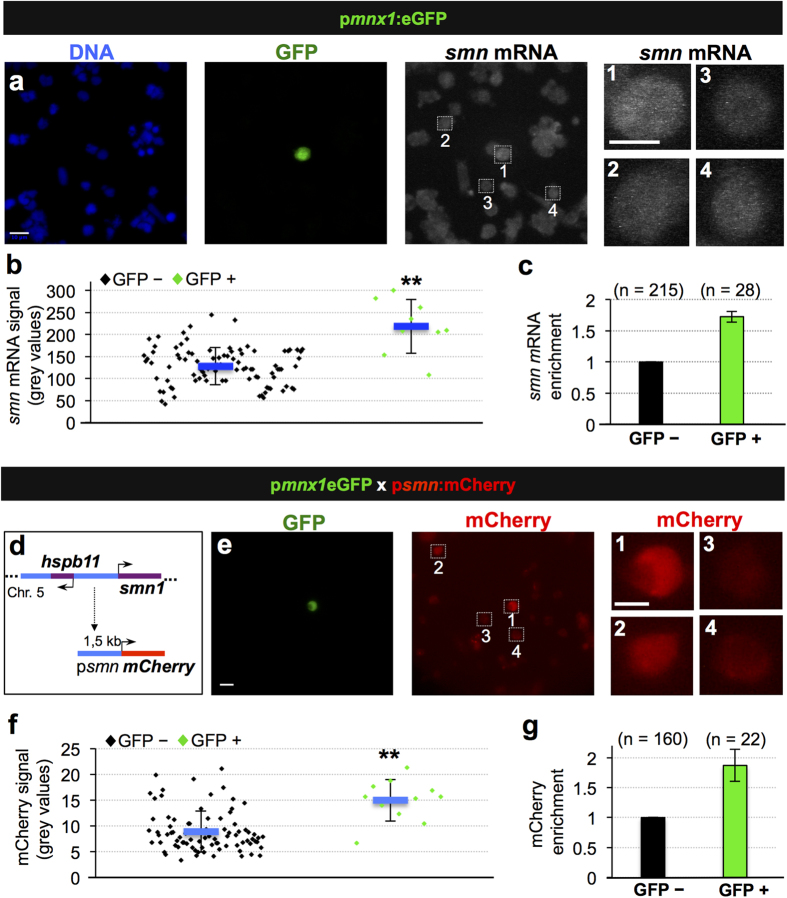
Transcriptional activation leads to increased Smn levels in motoneurons. (**a**) Single molecule FISH on p*mnx1*:eGFP embryos. DNA, GFP and *smn* mRNA signals are shown in Z-projected confocal sections. Scale bars: 10 μm for low and 5 μm for high magnification. (**b**) Quantification of *smn* mRNA signal from one representative experiment. Exact values are (mean ± SD): 127 ± 42 (GFP−) and 218 ± 61 (GFP+), p = 8.06 × 10^−8^ with Wilcoxon Sum Rank Test. (**c**) Average increase of *smn* mRNA levels in motoneurons versus control cells. The exact value of enrichment is 1.73 ± 0.09 (mean ± SD). N = 3 experiments, n = number of analyzed cells. (**d**) Putative *smn* promoter in zebrafish. Genomic situation (top) and mCherry construct (bottom). Arrows denote start of transcription. (**e**) scIF on p*mnx1*:eGFP × p*smn*:mCherry embryos. GFP and mCherry signals are shown. Scale bars: 10 μm for low and 5 μm for high magnification. (**f**) Quantification of mCherry signal from one representative experiment. Exact values are (mean ± SD): 8.9 ± 4 (GFP−) and 15 ± 4 (GFP+), p = 9.37 × 10^−5^ with Wilcoxon Sum Rank Test. (**g**) Average increase of mCherry levels in motoneurons versus control cells. The exact value of enrichment is 1.87 ± 0.27 (mean ± SD). N = 2 experiments, n = number of analyzed cells.

What is the mechanism leading to higher Smn protein levels in motoneurons? To answer this question, we first quantified *smn* mRNA levels in motoneurons and other cell types using single molecule FISH (Fig. 2a) and found that *smn* mRNA is enriched in motoneurons to an extent similar to Smn protein (Fig. 2b,c, compare with Fig. 1f). To explore whether this increase at mRNA level is due to differential *smn* promoter activity, we created a transgenic zebrafish line, in which the putative zebrafish *smn* promoter drives expression of mCherry (p*smn*:mCherry, Fig. 2d). Quantification of mCherry levels established that the *smn* promoter is more active in p*mnx1*:eGFP positive motoneurons compared with neighboring cells (Fig. 2e–g). Suggestively, the activity of the murine Smn promoter is also elevated in cultivated cells derived from the spinal cord when compared with fibroblasts^27^, raising the possibility that the motoneuron-specific transcriptional activation of *smn* described here in zebrafish is evolutionary conserved.

**Figure 3:**
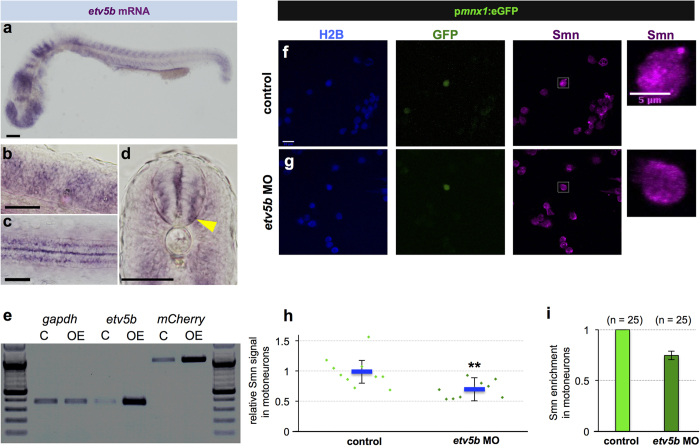
Etv5b activates the *smn* promoter leading to increased Smn levels in motoneurons. (**a–d**) Whole-mount *in situ* hybridization of *etv5b* expression in 24 hpf embryos. Expression pattern in the entire embryo (**a**), in magnified lateral (**b**) and dorsal (**c**) views as well as in a transverse section (**d**) of the trunk. In the latter case, yellow arrowhead denote signal in the motoneuron region. Scale bars: 100 μm (**a**) and 50 μm (**b–d**). nc, notochord. (**e**) RT-PCR in control (C - only p*smn:*mCherry) versus *etv5b* overexpressing (OE - *etv5b* mRNA and p*smn:*mCherr*y*) embryos at early gastrula stage. (**f**,**g**) scIF of control (**f**) and *etv5b* MO (**g**) cells from p*mnx1*:eGFP embryos. H2B, GFP and Smn signals are shown in Z-projected confocal sections. GFP+ cells marked by white rectangles are magnified on the right. Scale bars: 10 μm for low and 5 μm for high magnification. (**h**) Relative Smn signal in control and in *etv5b* MO cells from one representative experiment. Exact values are (mean ± SD): 0.99 ± 0.25 (control) and 0.7 ± 0.14 (*etvb5* MO), p = 0.003 with Wilcoxon Sum Rank Test. (**i**) Average relative Smn levels in control and in *etv5b* MO motoneurons. The exact value of decrease is 0.75 ± 0.04 (mean ± SD). N = 3 experiments, n = number of analyzed cells.

Next, we sought to identify the transcription factor driving additional *smn* transcription in motoneurons. The human, the murine and the zebrafish *smn* promoters contain several ETS transcription factor binding sites^27^,^28^ (Fig. S3a), and a member of this family, Pea3, is necessary for axon branching of motoneurons in mice^29^. Among the four Pea3 subfamily members in zebrafish (*etv1*, *etv4*, *etv5a*, *etv5b*)^30^,^31^, only *etv5b* shows a marked expression in the spinal cord (Fig. 3a–d and ZFIN database^32^). We therefore asked whether Etv5b is able to activate the *smn* promoter. Co-injection of *etv5b* mRNA and the p*smn:*mCherry plasmid lead to a robust increase of *mCherry* mRNA (Fig. 3e) and protein (Fig. S3b–g) in early embryos. This elevation was dependent on the two proximal ETS binding sites in the *smn* promoter (Fig. S3h,i). Apart from Etv5b, Etv4 was also able to activate the *smn* promoter (Fig. S4a) likely due to the high degree of conservation of the DNA-binding ETS domains present in this subfamily (Fig. S4b). Notably, *etv5b* may be controlling *smn* mRNA levels also in other cells and additional mechanisms may be at play in motoneurons. Altogether, our findings raised the possibility that Etv5b, acting in concert with unknown co-factor(s) present in motoneurons, is responsible for increased Smn levels in motoneurons. To test this, we knocked down *etv5b* as described previously^33^ (see also Fig. S5), and measured Smn protein levels in motoneurons. Importantly, unlike treatment with control morpholino (MO) (Fig. S2f), *etv5b* MO lead to a significant reduction of Smn in motoneurons (Fig. 3f–i), suggesting that Etv5b is needed for the additional Smn supply in motoneurons.

**Figure 4:**
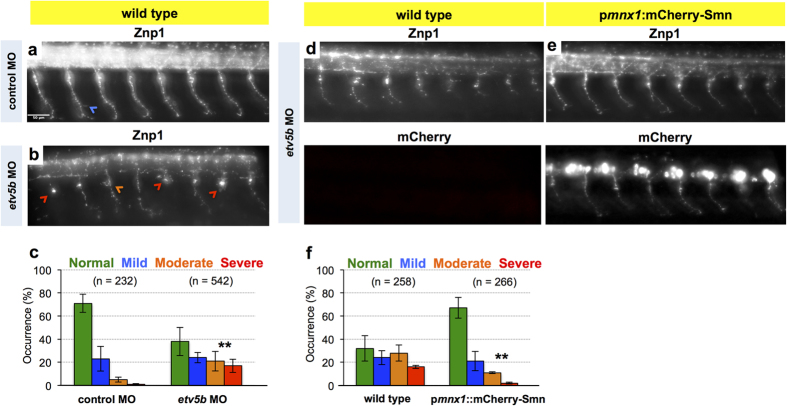
Knockdown of Etv5b results in axon defects due to motoneuronal reduction of Smn. (**a**,**b**) Z-projections of Znp1 stained motor axons on one side of an embryo after injection of control MO (**a**) and *etv5b* MO (**b**). Scale bar: 50 μm. Arrowheads mark different defects. (**c**) Quantification of axon defects. Normal, mild, moderate and severe axon morphology in control MO and *etv5b* MO injected embryos. N = 2 experiments (control MO, in total of 17 embryos) and 5 experiments (*etv5b* MO, in total of 42 embryos), n = number of axons analyzed. Significance is determined by comparing the occurrences of moderate and severe defects. Exact values in percentages of affected axons are (mean ± SD): 71 ± 7.8 (control MO, normal, 23 ± 10.6 (control MO, mild), 5 ± 2.1 (control MO, moderate), 1 ± 0.7 (control MO, severe), 38 ± 12.2 (*etv5b* MO, normal), 24 ± 4.4 (*etv5b* MO, mild), 21 ± 8.5 (*etv5b* MO, moderate) and 17 ± 5.8 (*etv5b* MO severe), p = 0.001 with unpaired Student’s t-test. (**d,e**) Z-projection of Znp1 stained motor axons on one side of control (**d**) and p*mnx1*:mCherry-Smn (**e**) embryos injected with *etv5b* MO. mCherry signal is shown below the Znp1 panel. (**f**) Quantification of axon morphology phenotypes in control and p*mnx1*:mCherry-Smn (mCh-S) embryos injected with *etv5b* MO. N = 3 experiments, n = number of axons analyzed in total of 22 (control MO) and 20 embryos (*etv5b* MO). Significance is determined by comparing the occurrences of moderate and severe defects. Exact values in percentages of affected axons are (mean ± SD): 32 ± 11.3 (control *etv5b* MO normal), 24 ± 5.9 (control *etv5b* MO mild), 28 ± 7 (control *etv5b* MO moderate), 16 ± 1.4 (control *etv5b* MO severe), 67 ± 9 (mCh-S *etv5b* MO normal), 21 ± 8.3 (mCh-S *etv5b* MO mild), 11 ± 0.8 (mCh-S *etv5b* MO moderate), 2 ± 0.9 (mCh-S *etv5b* MO severe), p = 0.009 with unpaired Student’s t-test.

Is the extra pool of Smn essential for motoneurons? We addressed this major point by analyzing motor axon morphology following *etv5b* MO treatment and found prominent branching and truncation defects (Fig. 4a–c). Of note, these defects are less pronounced than those in *smn* morphants^13^,^14^, which is in agreement with Smn not being completely absent from motoneurons following *etv5b* MO knockdown. To explore whether the defects observed upon *etv5b* knockdown arise in a cell-autonomous fashion (that is due to lower Smn levels in motoneurons), we performed the same experiment in p*mnx1*:mCherry-Smn embryos, which express Smn exclusively in motoneurons (Fig. 4e). In such *etv5b* MO injected embryos, the axon defects were ameliorated (Fig. 4d–f). Thus, the motoneuron-specific restoration of Smn levels is sufficient to suppress defects caused by *etv5b* knockdown, thereby establishing that elevated Smn levels in motoneurons are of functional relevance.

**Figure 5:**
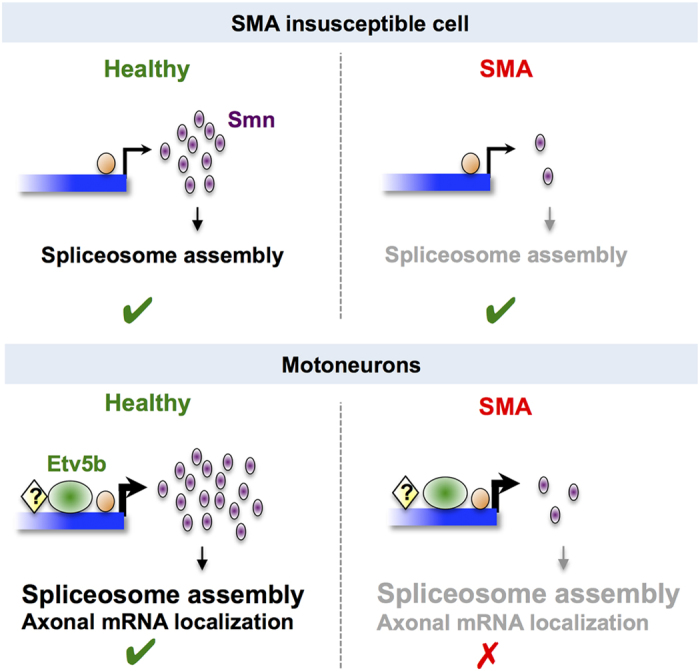
Model. Under healthy conditions in motoneurons, basal *smn* transcription (orange circle) is enhanced by Etv5b (green oval) acting together with other unknown factor(s) (yellow diamond) leading to more Smn protein (purple) in those cells. More Smn could be needed in motoneurons to meet an increased demand for the splicing machinery and/or for axonal mRNA localization. In the event of reduced Smn levels (that is in SMA), SMA-insusceptible cells are able to tolerate such a reduction, while motoneurons suffer from the cumulative reduction of multiple processes, thereby rendering them more vulnerable.

In conclusion, our data support a hypothesis where a transcriptional mechanism ensures elevated Smn levels in motoneurons, potentially due to a higher demand for Smn in those cells. We uncovered that this extra pool of Smn is essential for the proper axonal morphology of motoneurons in a cell-autonomous manner. Conceptually, these findings can explain why the reduction of Smn in SMA leads to more profound defects in motoneurons compared with other cells (see model in Fig. 5). Interestingly, normal human motoneurons were reported to express lower levels of full-length SMN from the SMN2 locus when compared to other cells^34^. This was explained by inefficient exon 7 inclusion in SMN2 particularly in this cell type and suggests that distinct mechanisms might act together to cause increased motoneuron vulnerability.

## Methods

### Generation and maintenance of transgenic zebrafish lines

All experiments were performed in accordance with relevant guidelines and regulations, and approved by the National University of Singapore (NUS) Institute of Animal Care and Use Committee (IACUC) (protocol numbers 082/10; R13-5714; BR15-0119). The 2.6 kb *mnx1* promoter was kindly provided by Dirk Meyer (University of Innsbruck)^35^. Transgenic lines were generated using I-Sce I meganuclease as previously described^36^. In the p*mnx1*:eGFP construct, eGFP was cloned with a Kozak sequence (GCCACC) right after the *mnx1* promoter. To generate the p*mnx1*:*mCherry*-*smn* construct, *mCherry* was cloned to the 5′ end of the *smn* cDNA via a flexible (SGGG)_3_ linker and subcloned into the pBSKII SK(+) plasmid. The zebrafish *smn* promoter was cloned by amplifying a 1547 bp region upstream of the start codon of *smn* (the DNA region between *smn* and the upstream gene, *hspb11*) with the following primer pairs:

Fwd: GCAGCATCCAGCATTTCGAG

Rev: GGGGATGTATGAAAATTTTAAATATTTCCC.

Subsequently, the p*smn:mCherry* construct was subcloned into the pBSKII SK(+) plasmid. The ΔETSp*smn*:GFP plasmid was generated by modifying the p*smn*:mCherry plasmid with swapping the wild type *smn* promoter with the mutated version of the promoter and mCherry with GFP, respectively. The mutated ETS sites are as follows: GGAA –> TTTT and ATCC –> TTTC, see also Fig. S3G. The p*nkx2.2a*:GFP line was generated in Bruce Appel’s lab (University of Colorado, Denver)^26^.

### Morpholino knockdown, RT-PCR and capped mRNA transcription

MOs were obtained from GeneTools. Control standard MOs were utilized as negative control, the anti-*smn* MO was used as described in^17^. The splice blocking anti-*etv5b* MO is described in^33^, and was used at a concentration of 125 μM.

To address the efficiency of the *etv5b* knockdown, RNA was isolated from 10–12 embryos at 24 hpf using the standard Trizol-Chloroform method followed by reverse transcription (RevertAid First Strand cDNA Synthesis Kit, Fermentas) with random hexamer primers. The exon spanning primer pairs amplifying the region between exon 10 and 11 were as follows:

*etv5b* exon 9–10 Fwd: GCAATGGTGAGGGTTTTGGG

*etv5b* exon 11–12 Rev: GCTCTCCAGCAACCTTTACC

In order to generate *etv5b* and *etv4* mRNA, both cDNAs were cloned into the pCS2+ plasmid after amplification with Phusion Taq polymerase using the following primer pairs:

*etv5b:*

Fwd: GAGAGTGCGATACCCACTCG

Rev: TCCATTTATGGATGTTTATTTATCCTGTACC.

*etv4:*

Fwd: TGAAGCAAAGAAGAGGTCTCGAGC

Rev: AGCAATCTCTTGAACCACAGTATTGTG

SP6-driven mRNA transcription was performed with the mMESSAGE mMACHINE kit (Ambion) for 2 hours at 37 °C. Thereafter, A-tailing was achieved by the Poly(A) Tailing Kit (Applied Biosystems) and the resulting mRNA was injected at a concentration of 300 ng/μl into one-cell stage embryos together with the p*smn*:mCherry and ΔETSp*smn*:GFP plasmids at concentration of 50 ng/μl.

For semi-quantitative RT-PCR, 20 early gastrula stage embryos were collected for RNA isolation and half of the isolated total RNA was reverse-transcribed using random hexamer primers with the above-mentioned kit. 1 μl of the cDNA was amplified for 23 (*mCherry*), 25 (*etv5b* and *etv4*) and 30 (*gapdh*) cycles using the following primer pairs:

*gapdh* Fwd: GATTGCCGTTCATCCATCTT

*gapdh* Rev: TCCATTTCTCACAAACAGAGGA

*etv5b* Fwd: GCAATGGTGAGGGTTTTGGG

*etv5b* Rev: GCTCTCCAGCAACCTTTACC

*etv4* Fwd: CCCCTCAGGAATCTGAAGACCTC

*etv4* Rev: TCTTCTGCTCATAGGCACTGGC

mCherry Fwd: CCACC ATGGTGAGCAAGGG

mCherry Rev: GCGAATTAAAAAACCTCCC

### Embryo dissociation, single cell immunofluorescence (scIF) and immunostaining on spinal cord sections

24 hpf embryos were dechorionated followed by deyolking in Ringer’s solution and then dissociated to single cells using a Papain Dissociation System Kit (Worthington) according to the manufacturer’s instructions. The dissociation was performed in a thermoshaker at 1100 rpm set to 30 °C for 30 minutes, with pipetting the mixture 3–4 times every 10 minutes to promote dissociation. Following the reconstitution of cells in Leibovitz medium containing 2% FBS, the cells were counted in a BioRad TC20 machine. 150,000 cells in 50 μl were plated on poly-L-lysine slides (Fisherbrand, for a 3-hour culture) or on Laminin-coated coverslips (Neuvitro, for a 24-hour culture) and incubated at 30 °C. Of note, when working on slides, control and treated cells were plated in droplets on opposing ends of the same slide well-separated from each other to minimize artifacts in subsequent steps arising from slide-to-slide variances. When the cells appeared attached to the surface (approximately after 1 hour of incubation), 200 μl (for slides) or 1 ml (for coverslips in 12-well plates) of culture medium (described in^37^) was added.

Following incubation, cells were washed twice in PBS in a 50 ml glass container (for slides) or in a 12-well plate (for coverslips) and then fixed in 4% PFA (Sigma) in PBS for 5 minutes at room temperature. Following a PBS wash, the cells were submerged into PBS containing 0.1% Triton-X (Sigma) for 5 minutes at room temperature. Blocking was performed by PBS/0.1% Triton-X containing 5% BSA for 2 hours at room temperature. The primary antibodies α-Smn (Novus Biological, NB-1001936 or NB100-1936AF647 1/100) and α-Histone2B (LifeSpan BioSciences, LS-B2035, 1/100) were diluted in PBS and applied in 50 μl/coverslip or 100 μl/slide and incubated at 4 °C overnight. The cells were washed 3 times with PBS/0.1% Tween-20 (PBS-T, Tween 20 from Sigma) for 5 minutes each at room temperature followed by a final wash in PBS for 5 minutes at room temperature. The secondary antibodies Alexa-633-conjugated α-mouse (Life Technologies, A21052, 1/500) and Alexa-405-conjugated α-rabbit (Life Technologies, A31556, 1/500) were diluted in PBS and applied in 50 μl/coverslip and 100 μl/slide, then incubated at room temperature for 2–4 hours. The cells were washed 3 times with PBS-T for 5 minutes each at room temperature followed by a final wash in PBS for 5 minutes at room temperature and mounted in Mowiol (Sigma). In experiments with DNA staining, DAPI was added to the final PBS-T wash at a concentration of 1 μg/ml.

The slides were imaged using a Leica LSM510 confocal microscope with 1.2 μm thick optical slices. We utilized laser intensities and digital gain that did not cause oversaturation of the signal measured by a range indicator built in the Zen 2009 software, where oversaturated pixels appear red. Thereafter, the same setup was used for all images of an experiment to avoid oversaturation. Maximum intensity projections were performed and the signal intensities were measured by manually drawing the boundaries of a given cell using ImageJ. Typically, the signal intensities of a motoneuron with strong *gfp* expression (that are considered primary motoneurons^38^) and 8–10 neighboring cells were quantified per field. In experiments where Smn levels were quantified relative to H2B, we divided the Smn grey values with those of H2B. When pooled values are displayed, the ratios of control vs. treated values from each set of experiment were taken and averaged (with control values being 1) thereby correcting for experiment-to-experiment variation.

Cell size measurements were performed with Image J by quantifying the total cell areas on the DIC channel of Z-projected confocal sections. Typically, the cell area of one *gfp* positive cell and 8–10 neighboring cells were quantified per field.

In experiments with the p*smn*:mCherry line, mCherry expression in fixed cells was recorded using a Nikon Eclipse 90i fluorescent microscope equipped with a Nikon DS Ri1 camera in the middle plane avoiding apparent oversaturation of the signal and the fluorescent intensities were quantified with ImageJ. The intensities of the background (i.e. areas on the slide void of cells) were subtracted from the intensities measured in cells.

Immunostaining of *in vivo* motoneurons in 24 hpf p*mnx1*:eGFP embryos (fixed in 4%PFA for 4 hours at room temperature) was performed on 16 μm thick sections of the posterior trunk using a standard cryo-sectioning protocol. The sections were dried for 1 hour at 37 °C, washed with PBS-T for 5 minutes at room temperature and permeabilized in PBS containing 0.5% Triton-X for 20 minutes at room temperature. Thereafter, the slices were fixed and processed the same way as described above for scIF and 3 μm thick optical slices were imaged with the LSM510.

### Whole-mount *in situ* hybridization and single molecule FISH

The whole-mount *in situ* hybridization was performed as previously reported^39^. The following primer pairs were used to clone a 500 bp long fragment of *etv5b* cDNA into the pCS2+ vector for riboprobe generation:

Fwd: AAAACTCCACCCTCCTGCTTCAGC

Rev: AGTCAAAGCCCACCGTTTGC

Fluorescent Quasar 570-conjugated probes against *smn* mRNA were designed and purchased from Biosearch Technologies and were used at a concentration of 1 μM. Dissociated cells grown on coverslips were fixed and probed with smFISH probes following the protocol for adherent cells provided by Biosearch Technologies (https://www.biosearchtech.com/assets/bti_stellaris_protocol_adherent_cell.pdf). The *smn* probes together with the mouse anti-GFP antibody (1:200, Invitrogen) were incubated at 37 °C overnight. The anti-mouse Alexa 488 conjugated secondary antibody (A11029, Life Technologies) was diluted in Wash buffer A in 1:1000 and incubated for 45 minutes at 37 °C.

The cells were imaged with an LSM510 confocal microscope using the 543 nm laser with open pinholes and optical sections of 2 microns avoiding oversaturation of the signal similarly as mentioned above. Maximum Z projections followed by manual drawing the boundaries of a cell and quantification of fluorescent intensities were performed with ImageJ. The intensities of the background (i.e. areas with no cells) were subtracted from the intensities measured in cells.

### Whole-mount immunofluorescence

The immunofluorescence was analyzed after standard PFA-methanol fixation. Embryos were incubated with the following primary antibodies: mouse α-ZNP-1 (1/100, ZIRC) and rabbit α-RFP (1/100, MBL, PM005); and with the following secondary antibodies: Alexa-568-conjugated α-rabbit (1/500, A11036, Life Technologies) and Alexa-488-conjugated α-mouse (1/500, A11029, Life Technologies) both at 4 °C overnight.

The embryos were imaged with a Nikon Eclipse 90i fluorescent microscope equipped with a Nikon DS Ri1 camera using 10 μm thick optical sections. The axon defects were assessed qualitatively as mild (a branching defect occurring at the last third of the axon), moderate (branching and/or truncation at the middle third of the axon) and severe (branching and/or truncation at the first third of the axon). Typically, 6–7 axon pairs above the yolk extension were examined in each embryo.

### Western blot and pre-adsorption experiments

10–30 dechorionated 24 hpf embryos were deyolked in Ringers solution and following centrifugation, the embryos were lysed in standard RIPA buffer on ice in a volume of 2 μl/embryo. Sample buffer was added, and the lysate was heated up at 95 °C for 5 minutes and loaded right away or stored at −20 °C. Following electrophoresis in a 12% poly-acrylamide gel, the proteins were transferred to PVDF membrane using wet transfer. Blocking was performed with 5% milk powder (Fluka) in TBS containing 0.1% Tween-20 (TBS-T) for 1 hour at room temperature. Primary antibodies were the same as the ones used in the scIF protocol with dilutions of 1:250 (α -Smn) and 1:500 (α -H2B). Secondary antibodies (Sigma) were applied in 1:5000 for 2 hours at room temperature. The chemiluminescent detection was performed with the SuperSignal West Pico Kit (Thermo scientific).

The pre-adsorption experiments were performed using a protein containing the first 160 amino acids of human SMN in complex with His-tagged GEMIN2 (referred to as antigen; a kind gift from Utz Fischer, University of Wuerzburg). We made use of Ni-NTA columns (Qiagen) following the manufacturer’s instructions. After equilibration with PBS, 300 μg of the antigen diluted in PBS was bound to the column for 1 hour at 4 °C. A control column was incubated with PBS void of antigen. After washing with PBS, 5 μg of the antibody diluted in PBS was applied to the column and incubated overnight at 4 °C. To ensure efficient binding of the antibody to the antigen, the antibody solution was spun through the column three times during this incubation. Thereafter, we used the flow-through for Western blot and scIF.

### Statistical analysis

Experiments were performed in triplicates, unless otherwise stated. Statistical analysis of all cell culture experiments was performed using Wilcoxon Sum Rank Test (also known as Mann-Whitney *U* Test), while unpaired Student’s t-test was used in the case of the axon morphology experiments, both executed in Excel. Pooled data are displayed as mean ± SD with significance values of *p < 0.05 and **p < 0.001. All values and significance measurements used in this study are displayed in Supplementary Table S1.

## Additional Information

**How to cite this article**: Spiró, Z. *et al.* Transcriptional enhancement of Smn levels in motoneurons is crucial for proper axon morphology in zebrafish. *Sci. Rep.* **6**, 27470; doi: 10.1038/srep27470 (2016).

## Supplementary Material

Supplementary Information
