# Supplementary material for: Transcriptional enhancement of Smn levels in motoneurons is crucial for proper axon morphology in zebrafish
Source: Sci Rep. 2016 Jun 7;6:27470. doi: 10.1038/srep27470 (PMC4895340; doi:10.1038/srep27470)
Supplement: Supplementary Information [file srep27470-s1.pdf]

Supplementary Information

Transcriptional enhancement of Smn levels in motoneurons is crucial for proper axon morphology in zebrafish

Zoltán Spiró[1,2], Angela Koh[1,2], Shermaine Tay[1,2], Kelvin See[1,2,3] and Christoph Winkler[1,2]

Supplemental Figure Legends

Figure S1

Addressing the specificity of the Smn antibody and motoneuronal Smn levels *in vivo*

a-c scIF of wild type (a) and *smn* MO (b) cells as well as of cells without primary Smn-antibody (c). DNA (blue) and Smn (purple) are shown. Scale bar: 10 µm.

d Smn signal in control (black) and in *smn* MO (dark grey) cells as well as in cells without Smn-antibody (light grey) from a representative experiment. Exact values are (mean ± SD): 445 ± 206 (control), 204 ± 71 (*smn* MO) and 105 ± 44 (background), $p = 2.95 \times 10^{-13}$ with Wilcoxon Sum Rank Test.

e Average relative Smn levels in control (black) and in *smn* MO (dark grey) cells. N = 2 experiments. , n = number of analyzed cells. Exact value of decrease is (mean ± SD): 0.32 ± 0.04.

f Western blot analysis of Smn levels of control and of Smn MO-treated embryos. Arrowhead marks the band at the expected molecular weight of Smn (38 kDa), asterisk denotes an unspecific band. Histone 2B (H2B) was used as loading control.

g Pre-adsorption performed with the purified N-terminal half of human SMN1. Following pre-adsorption, 100 ng of the N-terminal part of SMN1 was blotted in duplicates (1. and 2.) with control ('no antigen' on the column while pre-adsorbing, upper left image) or pre-adsorbed ('antigen', upper right image) antibody solution. The same membranes were blotted thereafter with untreated antibody proving the presence of SMN1 on the both membranes (lower images).

h-i ScIF performed with control (h) and pre-adsorbed (i) antibodies in one representative experiment. Histone 2B (blue) and Smn (purple) are shown. Scale bar: 10 µm.

j Smn levels relative to H2B of the pre-adsorption experiment shown in h and i. Exact values are (mean ± SD): 1.36 ± 0.4 (control), 0.22 ± 0.02 (pre-adsorbed), $p = 1 \times 10^{-13}$ with Wilcoxon Sum Rank Test.

k Average relative Smn levels in control (black) and in pre-adsorbed (grey) cells. N = 2 experiments. , n = number of analyzed cells. Exact value of decrease is (mean ± SD): 0.11 ± 0.07.

l Smn immunostaining on cryo-sectioned spinal cord from a 24 hpf p*mnx1*:eGFP embryo. Z-projection with DNA (blue), GFP (green) and Smn (purple) is shown. The blue dashed rectangle labels the area where a dorso-ventral intensity scan was performed with Image J, which is displayed next to the Smn image; blue arrows mark the location of the motoneuron. The area marked with white dashed rectangle on the Smn image is magnified on the very right. Several sections from seven embryos were analyzed; a representative section is shown. Scale bars are 10 µm and 5 µm. Of note, as motoneuron axons contain considerable amount of Smn, its levels in cell bodies are an underestimation of total Smn content, likely explaining why the enrichment is less clear *in vivo* compared with the *ex vivo* setup, where the axons are retracted.

Figure S2

Smn levels are not higher in motoneurons due to a difference in cell size, are not elevated in perineurium cells and are not altered in motoneurons following control MO injection

a Cell area measurement in GFP negative (black diamonds) and GFP positive (green diamonds) cells of p*mnx1*:eGFP embryos from a representative experiment. The exact values are (mean ± SD): 30.4 μm^2 ± 4.8 (GFP -) and 30.1μm^2 ± 8.7 (GFP +), p = 0.48 with Wilcoxon Sum Rank Test.

b Average cell size of GFP negative (black) and GFP positive (green) cells. N = 3 experiments. n = number of analyzed cells. Exact values are (mean ± SD): 32.3 μm^2 ± 2.5 (GFP -) and 33 μm^2 ± 6.5 (GFP +).

c scIF on p*nkx2.2a*:GFP embryos. GFP and Smn signals are shown in Z-projected confocal sections.

d Quantification of absolute Smn signal from a representative experiment. Exact values are (mean ± SD): 400 ± 122 (GFP -) and 335 ± 100 (GFP +), p=0.06 with Wilcoxon Sum Rank Test.

e Ratio of Smn levels in perineurial cells (green) versus control cells (black). N = 3 experiments. n = number of analyzed cells. The exact value is (mean ± SD): 0.89 ± 0.05.

f Quantification of scIF performed on p*mnx1*:eGFP embryos in wild type and in control MO cells. Exact values are (mean ± SD): 0.83 ± 0.17 (GFP -) and 0.78 ± 0.19 (GFP +), p=0.46 with Wilcoxon Sum Rank Test.

Figure S3

Evolutionary conservation of proximal ETS-binding sites in the *smn* promoter and overexpression and knock-down of *etv5b*

a The two proximal ETS binding sites (with consensus sites being GGAA/T or A/TTCC) shown in green in the human, murine and zebrafish *smn* promoters. Note that the binding sites in human and mouse are on the forward strands, while in zebrafish it is located in the reverse strand. The first 200 nucleotides upstream of the start codons are shown.

b-**g** mCherry immunostaining (white) at the gastrula stage following p*smn:mCherry* injection without (b-d) or with Etv5b overexpression (e-g) on the same batch of embryos that was used for RT-PCR analysis shown in Fig. 3e. DNA is shown in blue. Scale bar: 100 microns. 8 embryos of control and 5 embryos with Etv5b overexpression were analyzed.

h Schematic representation of the first 40 nucleotides of the *smn* promoter with intact (p*smn*:mCherry, top) and mutated (ΔETSpsmn:GFP, bottom) ETS elements used in the experiment in panel i.

i A representative gastrula stage embryo that had been co-injected with Etv5b mRNA, the p*smn*:mCherry plasmid as well as the ΔETSp*smn*:GFP plasmid and immunostained against mCherry and GFP. DNA is shown in blue. 5 embryos showing strong mCherry staining were analyzed.

<u>Figure S4</u>

<u>**Etv4 is able to activate the *smn* promoter likely due to the conserved ETS domain in the Pea3 subfamily**</u>

<u>**a**</u> RT-PCR in control (C - only p*smn:*mCherry) versus *etv4* overexpressing (OE – *etv4* mRNA and p*smn:*mCherry) embryos at early gastrula stage.

b Sequence alignment of the four Pea3 subfamily members *etv1*, *etv4*, *etv5a* and *etv5b* using the Clustal Omega algorithm[1,2]. Stars denote the conserved amino acids, colors mark small + hydrophobic -Y (red), acidic (blue), basic - H (purple), hydroxyl + sulfhydryl + amine + G (green) and unusual (grey) amino acids. A red line on the right side indicates the approximate location of the C-terminal ETS domain. The sequences were retrieved from Ensembl (Release 83).

Figure S5

Cartoon explaining the action of the splice-blocking *etv5b* MO. The MO leads to a retention of intron 10 in *etv5b* mRNA that can be revealed by RT-PCR (right) by primers binding to exon 9 and exon 11 (black arrows on the mRNA). The gel shows a size shift of the amplicon in *etv5b* morphants.

Supplementary Table S1

Values and statistical significances of all data presented in this study in the order of appearance. The type of experiment, the number of biological replicates with reference to the figure panel where the given data is presented, the observed cell types or conditions, the number of cells or axons measured, the mean ± SD and the *p*-values calculated by Wilcoxon Sum Rank Test (cell culture experiments) and unpaired Students' t-tests (axon morphology experiments) are shown. Absolute signal intensities denote grey values measured by ImageJ, while relative Smn signal intensities indicate ratio of Smn grey values and the H2B grey values.

Supplemetal Table S1

experiment	N (experiments)	cell type	n (cells)	measurement	mean±SD	p-value
scIF on *pmnx1*:eGFP	**replicates**					
	1 (Fig. 1e)	GFP -	58	relative Smn signal intensities	0.51 ± 0.14	<0.01
		GFP +	9		0.87 ± 0.17	
	2	GFP -	54		0.27 ± 0.11	<0.01
		GFP +	9		0.46 ± 0.06	
	3	GFP -	29		0.7 ± 0.16	<0.01
		GFP +	8		1.07 ± 0.15	
	**average ratio** (Fig. 1f)	GFP -	141		1	na
		GFP +	26		**1.67 ± 0.14**	
scIF on wild type embryos - *smn* MO	**replicates**					
	1 (Fig. S1d)	control	75	absolute Smn signal intensities	445 ± 206	<0.01
		smn MO	46		204 ± 71	
		background	39		105 ± 44	
	2	control	56		705 ± 171	<0.01
		smn MO	67		450 ± 139	
		background	81		318 ± 73	
	**average ratio** (Fig. S1e)	control	131		1	na
		smn MO	113		**0.32 ± 0.04**	
pre-adsorption on wild type embryos	**replicates**					
	1 (Fig. S1j)	control	36	relative Smn signal intensities	1.36 ± 0.4	<0.01
		pre-adsorbed	33		0.22 ± 0.02	
	2	control	37		1.42 ± 0.5	<0.01
		pre-adsorbed	36		0.09 ± 0.02	
	**average ratio** (Fig. S1k)	control	73		**1**	na
		pre-adsorbed	69		**0.11 ± 0.07**	
cell area measurements - *pmnx1*:eGFP	**replicates**					
	1 (Fig. S2a)	GFP -	40	cell area (square microns)	30.4 ± 4.8	0.48
		GFP +	7		30.1 ± 8.7	
	2	GFP -	38		28.1 ± 8.1	0.16
		GFP +	8		31.8 ± 5.1	
	3	GFP -	38		40.4 ± 4.6	0.06
		GFP +	8		35 ± 7.1	
	**average ratio** (Fig. S2b)	GFP -	116		**32.3 ± 2.5**	na
		GFP +	23		**33 ± 6.5**	

experiment	N (experiments)	cell type	n (cells)	measurement	mean±SD	p-value
scIF on p*nkx2.2a*:GFP	**replicates**					
	1 (Fig. S2d)	GFP -	75	absolute Smn signal intensities	400 ± 122	0.06
		GFP +	12		335 ± 100	
	2	GFP -	66		720 ± 166	0.14
		GFP +	10		666 ± 178	
	3	GFP -	69		740 ± 133	0.37
		GFP +	10		683 ± 93	
	**average ratio** (Fig. S2e)	GFP -	210		1	na
		GFP +	32		**0.89 ± 0.05**	
smFISH on p*mnx1*:eGFP	**replicates**					
	1 (Fig. 2b)	GFP -	91	absolute smFISH signal intensities	127 ± 42	<0.01
		GFP +	9		218 ± 61	
	2	GFP -	68		131 ± 47	<0.01
		GFP +	10		236 ± 58	
	3	GFP -	56		240 ± 78	<0.01
		GFP +	9		394 ± 72	
	**average ratio** (Fig. 2c)	GFP -	215		1	na
		GFP +	28		**1.73 ± 0.09**	
scIF on p*mnx1*:eGFP x p*smn*:mCherry	**replicates**					
	1 (Fig. 2f)	GFP -	90	absolute mCherry signal intensities	8.9 ± 4	<0.01
		GFP +	12		15 ± 4	
	2	GFP -	70		18.4 ± 6.8	<0.01
		GFP +	10		38 ± 10.2	
	**average ratio** (Fig. 2g)	GFP -	160		1	na
		GFP +	22		**1.87 ± 0.27**	
scIF on p*mnx1*:eGFP - *etv5b* MO	**replicates**					
	1 (Fig. 3h)	GFP + control	10	relative Smn signal intensities	0.99 ± 0.25	<0.01
		GFP + *etv5b* MO	10		0.7 ± 0.14	
	2	GFP + control	7		1.04 ± 0.12	<0.01
		GFP + *etv5b* MO	7		0.82 ± 0.16	
	3	GFP + control	8	absolute Smn signal intensities	583 ± 90	0.02
		GFP + *etv5b* MO	8		446 ± 134	
	**average ratio** (Fig. 3i)	GFP + control	25	rel. or abs. Smn signal intensities	1	na
		GFP + *etv5b* MO	25		**0.75 ± 0.04**	
scIF on p*mnx1*:eGFP - control MO	**replicates**					
	1 (Fig. S2f)	GFP + wild type	9	relative Smn signal intensities	0.83 ± 0.17	0.46
		GFP + control MO	8		0.78 ± 0.19	

experiment	N (experiments)	condition	n (axons)	measurement	% (axons)	p-value
motoneuron axon morphology - *etv5b* MO in wild type	**replicates**					
	1	control MO normal	64	axon morphology judged by ZNP-1 staining	65	
	1	control MO mild	30		31	
	1	control MO moderate	3		3	
	1	control MO severe	1		1	
	1	*etv5b* MO normal	76		55	
	1	*etv5b* MO mild	36		26	
	1	*etv5b* MO moderate	14		10	
	1	*etv5b* MO severe	12		9	
	2	control MO normal	102		76	
	2	control MO mild	22		16	
	2	control MO moderate	8		6	
	2	control MO severe	2		2	
	2	*etv5b* MO normal	48		33	
	2	*etv5b* MO mild	32		22	
	2	*etv5b* MO moderate	29		20	
	2	*etv5b* MO severe	37		25	
	3	*etv5b* MO normal	29		47	
	3	*etv5b* MO mild	10		16	
	3	*etv5b* MO moderate	12		19	
	3	*etv5b* MO severe	11		18	
	4	*etv5b* MO normal	32		24	
	4	*etv5b* MO mild	36		27	
	4	*etv5b* MO moderate	44		33	
	4	*etv5b* MO severe	20		15	
	5	*etv5b* MO normal	22		34	
	5	*etv5b* MO mild	16		25	
	5	*etv5b* MO moderate	16		25	
	5	*etv5b* MO severe	10		16	
	**pooled values** (Fig. 4c)	control MO normal	166		71 ± 7.8	
		control MO mild	52		23 ± 10.6	
		control MO moderate	11		5 ± 2.1	
		control MO severe	3		1 ± 0.7	
		etv5b MO normal	207		38 ± 12.2	
		etv5b MO mild	130		24 ± 4.4	
		etv5b MO moderate	115		21 ± 8.5	<0.01
		etv5b MO severe	90		17 ± 5.8	

experiment	N (experiments)	Condition	n (axons)	measurement	% (axons)	p-value
motoneuron axon morphology - *etv5b* MO in wild type and in p*mnx1*::mCherry-Smn (mCh-S)	**replicates**			axon morphology judged by ZNP-1 staining		
	1	control, *etv5b* MO normal	29		47	
		control, *etv5b* MO mild	10		16	
		control, *etv5b* MO moderate	12		19	
		control *etv5b* MO severe	11		18	
		mCh-S, *etv5b* MO normal	39		75	
		mCh-S, *etv5b* MO mild	6		12	
		mCh-S, *etv5b* MO moderate	6		12	
		mCh-S, *etv5b* MO severe	1		2	
	2	control, *etv5b* MO normal	32		24	
		control, *etv5b* MO mild	36		27	
		control, *etv5b* MO moderate	44		33	
		control *etv5b* MO severe	20		15	
		mCh-S, *etv5b* MO normal	91		69	
		mCh-S, *etv5b* MO mild	26		20	
		mCh-S, *etv5b* MO moderate	14		11	
		mCh-S, *etv5b* MO severe	1		1	
	3	control, *etv5b* MO normal	22		34	
		control, *etv5b* MO mild	16		25	
		control, *etv5b* MO moderate	16		25	
		control *etv5b* MO severe	10		16	
		mCh-S, *etv5b* MO normal	47		57	
		mCh-S, *etv5b* MO mild	23		28	
		mCh-S, *etv5b* MO moderate	10		12	
		mCh-S, *etv5b* MO severe	2		2	
	**pooled values** (Fig. 4f)	control, *etv5b* MO normal	83		32 ± 11.3	
		control, *etv5b* MO mild	62		24 ± 5.9	
		control, *etv5b* MO moderate	72		28 ± 7	
		control *etv5b* MO severe	41		16 ± 1.4	
		mCh-S, *etv5b* MO normal	177		67 ± 9	
		mCh-S, *etv5b* MO mild	55		21 ± 8.3	
		mCh-S, *etv5b* MO moderate	30		11 ± 0.8	<0.01
		mCh-S, *etv5b* MO severe	4		2 ± 0.9	

Supplementary References

1 Goujon, M. *et al*. A new bioinformatics analysis tools framework at EMBL-EBI. *Nucleic Acids Res* **38**, W695-699, doi:10.1093/nar/gkq313 (2010).

2 Sievers, F. *et al*. Fast, scalable generation of high-quality protein multiple sequence alignments using Clustal Omega. *Mol Syst Biol* **7**, 539, doi:10.1038/msb.2011.75 (2011).

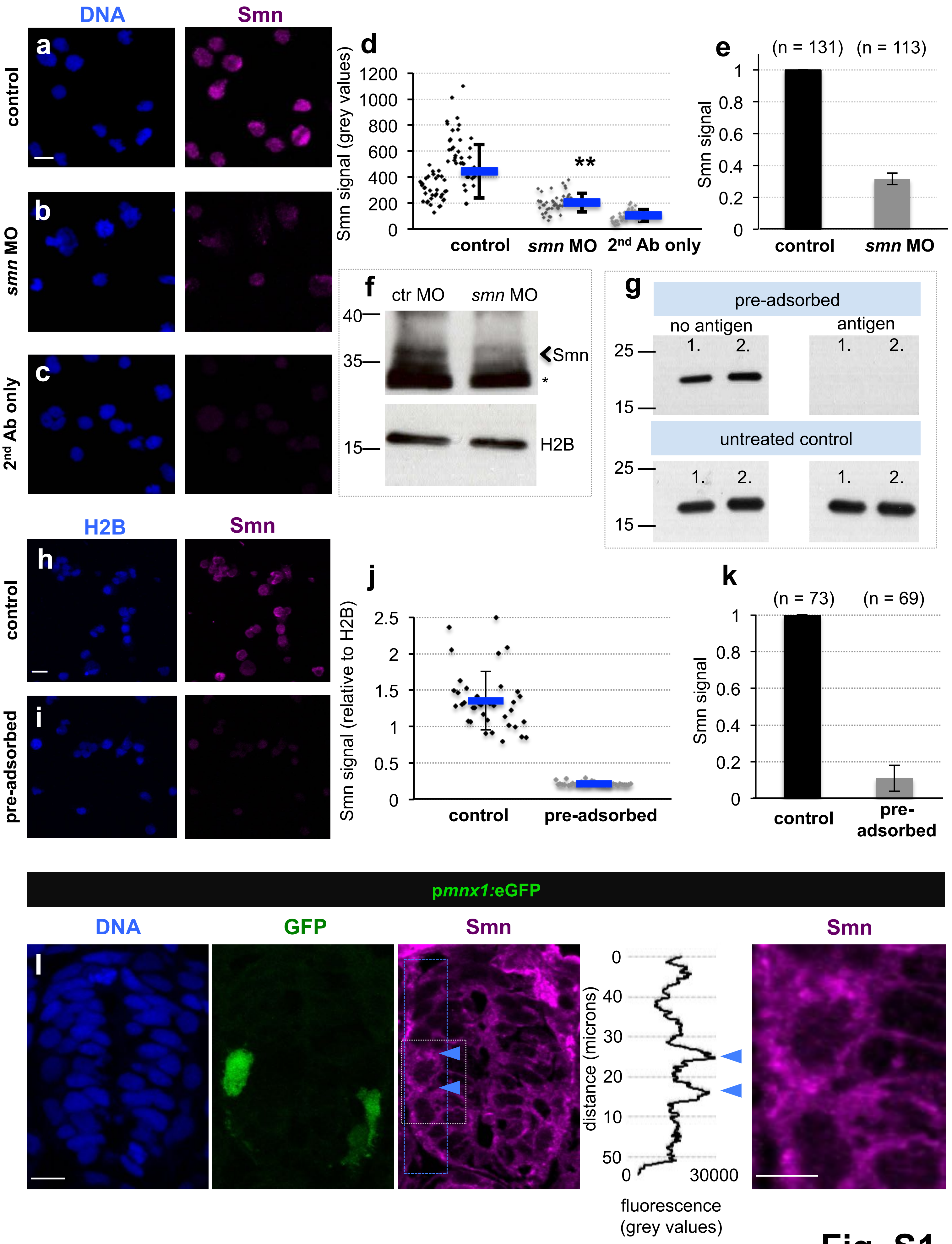

DNA
Smn
a
b
c
control
smn MO
2nd Ab only
d
Smn signal (grey values)
1200
1000
800
600
400
200
0
**
control
smn MO
2nd Ab only
e
(n = 131)
(n = 113)
Smn signal
1
0.8
0.6
0.4
0.2
0
control
smn MO
f
ctr MO
smn MO
40
35
15
Smn
*
H2B
g
pre-adsorbed
no antigen
antigen
1.
2.
25
15
untreated control
H2B
Smn
h
i
control
pre-adsorbed
j
Smn signal (relative to H2B)
2.5
2
1.5
1
0.5
0
control
pre-adsorbed
k
(n = 73)
(n = 69)
Smn signal
1
0.8
0.6
0.4
0.2
0
control
pre-adsorbed
pmnx1:eGFP
DNA
GFP
Smn
l
distance (microns)
0
40
30
20
10
50
0
30000
fluorescence (grey values)
Smn

Fig. S1

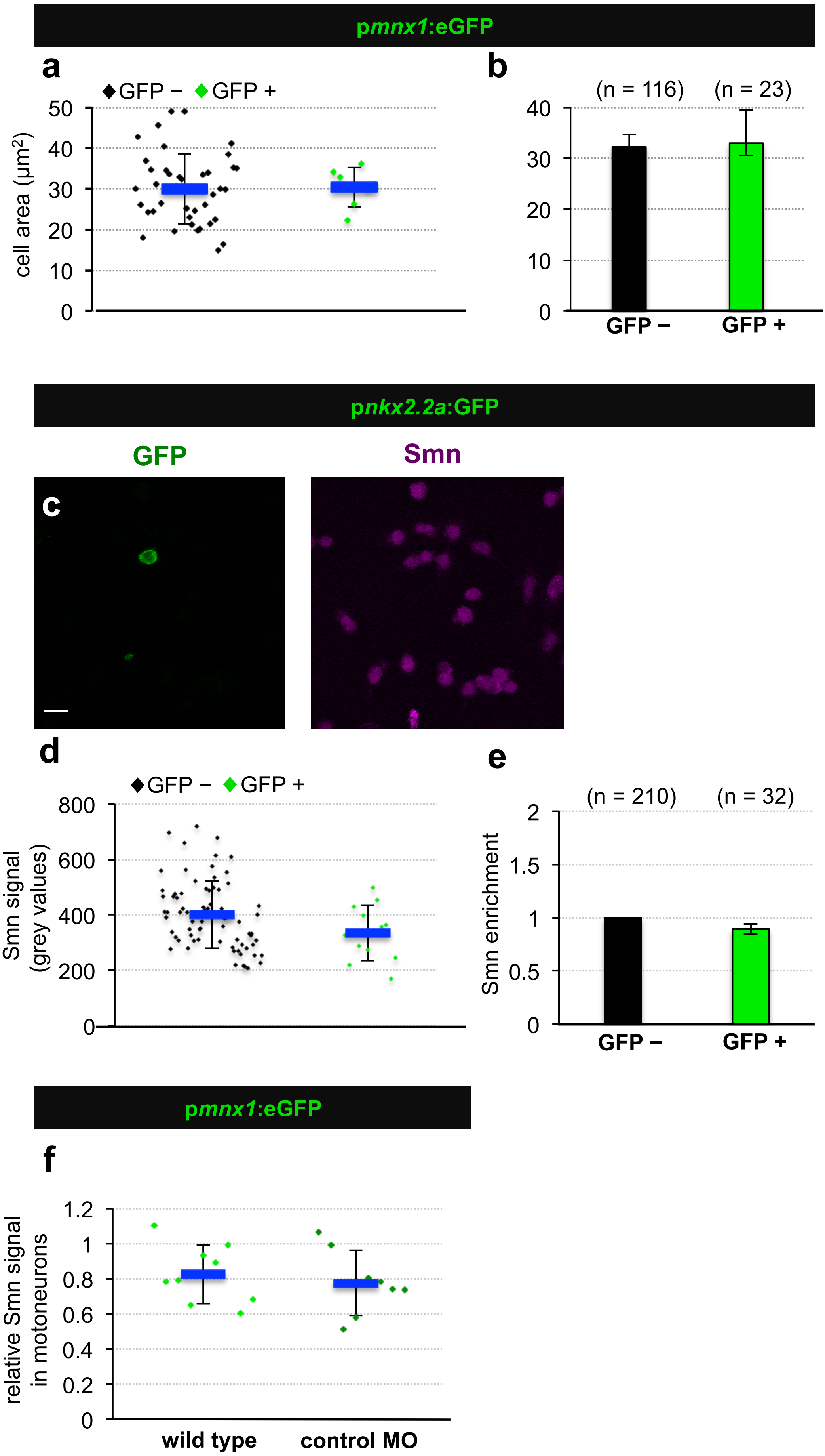

pmnx1:eGFP
a
GFP – GFP +
cell area (µm²)
50
40
30
20
10
0
b
(n = 116)
(n = 23)
40
30
20
10
0
GFP –
GFP +
pnkx2.2a:GFP
GFP
Smn
c
d
GFP – GFP +
Smn signal
(grey values)
800
600
400
200
0
e
(n = 210)
(n = 32)
Smn enrichment
2
1.5
1
0.5
0
GFP –
GFP +
pmnx1:eGFP
f
relative Smn signal
in motoneurons
1.2
1
0.8
0.6
0.4
0.2
0
wild type
control MO

Fig. S2

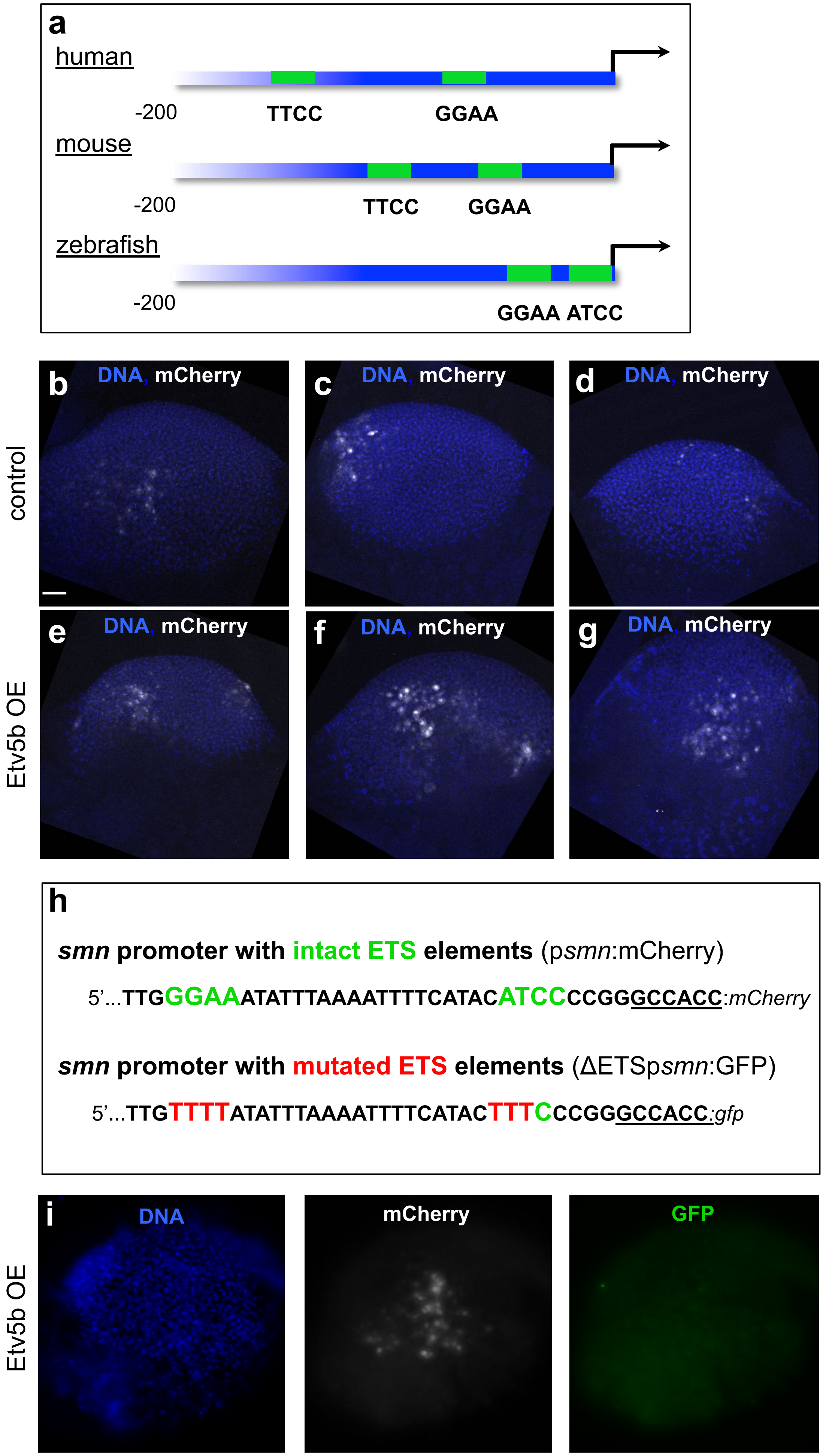
a
human
-200
TTCC
GGAA
mouse
-200
TTCC
GGAA
zebrafish
-200
GGAA ATCC
control
b DNA, mCherry
c DNA, mCherry
d DNA, mCherry
Etv5b OE
e DNA, mCherry
f DNA, mCherry
g DNA, mCherry
h
smn promoter with intact ETS elements (psmn:mCherry)
5'...TTGGGAAATATTTAAAATTTTCATACATCCCCGGGCCACC:mCherry
smn promoter with mutated ETS elements (ΔETSpsmn:GFP)
5'...TTGTTTTATATTTAAAATTTTCATACTTTCCCGGGCCACC:gfp
i
Etv5b OE
DNA
mCherry
GFP

Fig. S3

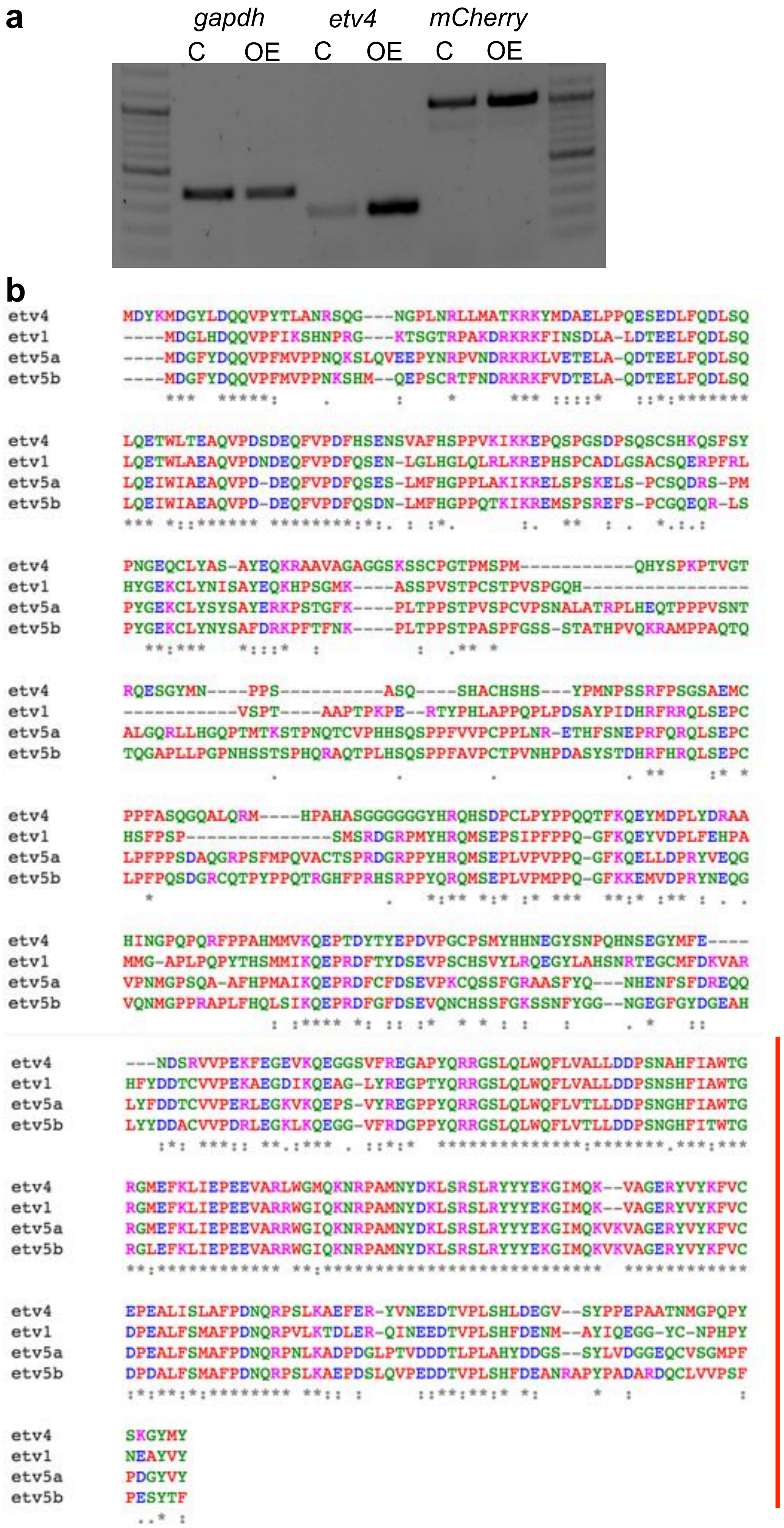

Fig. S4

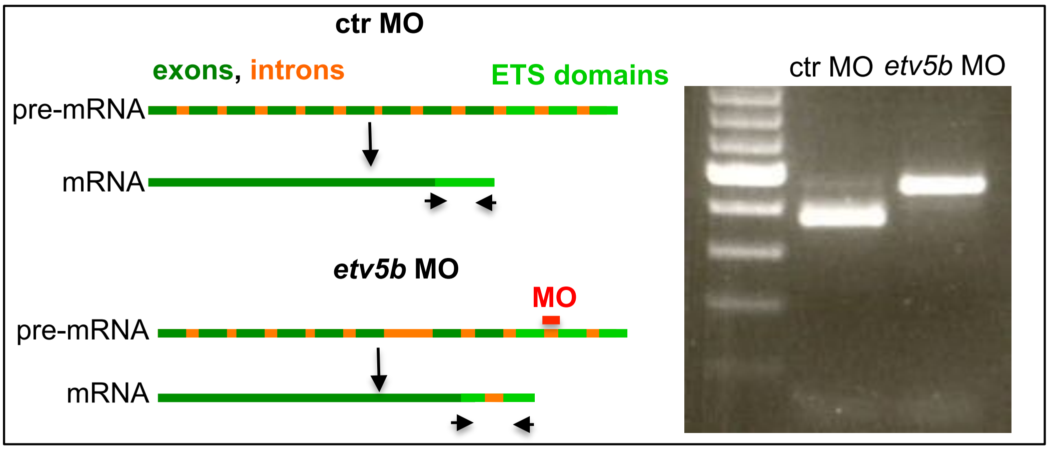
ctr MO
exons, introns
ETS domains
pre-mRNA
mRNA
etv5b MO
MO
pre-mRNA
mRNA
ctr MO
etv5b MO

Fig. S5
